# A Scoping Review of Glucose Spikes in People Without Diabetes: Comparing Insights from Grey Literature and Medical Research

**DOI:** 10.1177/11795514251381409

**Published:** 2025-10-25

**Authors:** Shira Avner, Timothy Robbins

**Affiliations:** 1 Warwick Medical School, University of Warwick, Coventry, UK; 2 Warwickshire Institute for the Study of Diabetes, Endocrinology and Metabolism (WISDEM), University Hospitals Coventry and Warwickshire NHS Trust, Coventry, UK

**Keywords:** continuous glucose monitoring, glucose spikes, glycaemic variability, insulin resistance, cardiovascular disease, non-diabetic

## Abstract

**Background:**

The use of continuous glucose monitoring (CGM) has grown, extending its use to people without diabetes. CGM helps prevent hyperglycaemia-related complications in diabetes, however, its value in people without diabetes remains uncertain. Despite online sources framing glucose spikes as harmful, studies show that overall, most healthy individuals maintain normal glucose levels – therefore questioning the significance of these spikes. This project aims to examine whether glucose spikes affect the health of people without diabetes. By comparing the medical and grey literature, we aim to determine whether the grey literature aligns with the peer-reviewed medical literature, or whether it could cause harm through misinformation.

**Methods:**

Population: people without diabetes and human endothelial cells; Concept: the effect of glucose spikes on health; Context: global studies and grey literature. Following the PRISMA-ScR guidelines, a systematic search was undertaken via Medline, Embase and Proquest. Fifty-nine sources were reviewed − 11 medical research papers and 48 grey literature sources. Excel spreadsheets were developed and piloted for the medical and grey literature respectively. Data was extracted and charted, and a narrative synthesis was formulated.

**Results:**

Both the medical and grey literature reported glucose spikes can cause endothelial dysfunction, oxidative stress and inflammation in people without diabetes or human endothelial cells. However, the grey literature reported additional effects that is, increased risk of cancer and effects on mental health, energy, mood and sleep.

**Conclusions:**

Glucose spikes may impact the health of people without diabetes, but significant health outcomes likely stem from long-term frequent spikes rather than isolated acute spikes. Discrepancies between the medical and grey literature highlight potential for misinformation in the grey literature, although the author does not claim cited sources are misleading, nor does the absence of claims in medical literature mean grey literature is misinforming. Further research is needed to verify if grey literature claims align with peer-reviewed evidence, as hypothetically, misinformation could significantly impact consumer wellbeing.

## Plain Language Summary

Evaluation of the effects of sudden increases in blood sugar (glucose spikes) in people without diabetes

Introduction Public awareness of glucose spikes has increased, and there have been developments in use of continuous glucose monitoring (CGM) in people without diabetes to monitor glucose spikes. Whilst CGM helps prevent diabetes complications like stroke and nerve damage, it is unclear whether CGM and controlling glucose spikes affect overall health in people without diabetes. There is little research published about glucose spikes in healthy people, and it is important to understand if CGM can be beneficial or whether it can cause unhealthy eating habits. This paper compares current research with information published in newspapers, magazines and blogs (grey literature). Methods A scoping review method was chosen to compare medical evidence with grey literature, to identify gaps in the knowledge and to make recommendations for future practice. Medline and Embase databases were searched using key terms. Proquest database was searched for grey literature. The data was extracted from the reviewed papers using an excel data extraction spreadsheet, then reviewed and summarised. Results Both the medical and grey literature reported that glucose spikes can affect blood vessel function, increase cell damage and inflammation in people without diabetes or human cells. However, the grey literature also links spikes to risks like cancer, mental health issues, and impacts on energy, mood, and sleep. Conclusion Glucose spikes may affect the health of people without diabetes. However, significant health problems are more likely to be from repeated glucose spikes rather than one-off spikes. Differences between the medical and grey literature suggest potential for misinformation, though we do not suggest grey sources in this paper are misinforming. Further research is needed to understand whether the additional claims in the grey literature have equivalent medical evidence as hypothetically the effect of misinformation on the public could be considerable.

## Introduction

In recent years, awareness of glucose spikes has increased and there have been developments in continuous glucose monitoring (CGM) for monitoring glucose levels both in people with and without diabetes. CGM is a medical technology which has been available since 2000^1^ used to measure interstitial fluid glucose levels, which reflects blood glucose concentration.^2^ There are known advantages in CGM for people with diabetes due to the high risk of complications from chronic hyperglycaemia. These include microvascular complications for example, neuropathy, retinopathy, and macrovascular complications for example, stroke.^3^

Diabetes mellitus is a metabolic disorder characterised by high glucose levels (hyperglycaemia) due to defects in insulin production, action or both. There are 2 major types of diabetes – type 1 diabetes is an autoimmune condition whereby pancreatic beta islet cells are destroyed, leading to complete insulin deficiency. Type 2 diabetes mellitus is characterised by insulin resistance or insufficient insulin production to meet physiological demand.^4^

In people without diabetes, blood glucose is tightly regulated within a narrow physiological range of 4.4 to 5 mmol/L under fasted conditions.^5^ Following a meal, glucose levels peak at approximately 1 hour and return to baseline within 2 to 3 hours.^6^

**Figure 1.**
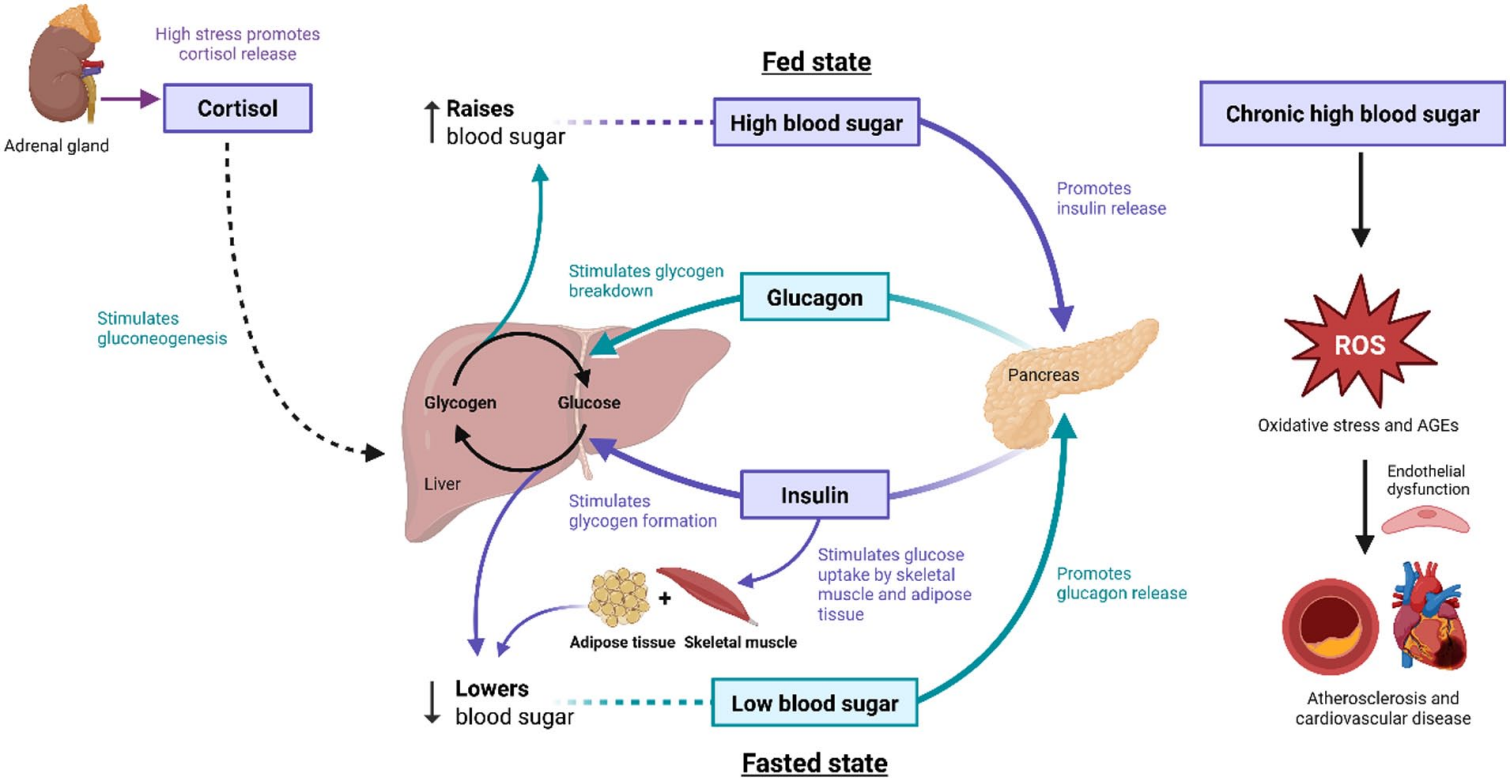
Diagram illustrating glucose homoeostasis. Overview of how key organs (pancreas, liver, skeletal muscle, adipose tissue, adrenal glands) regulate blood glucose during fed and fasted states. Hormonal control is mediated primarily by insulin, glucagon and cortisol. Disruption of this balance can lead to oxidative stress, advanced glycation end-products (AGEs) and endothelial dysfunction, contributing to cardiovascular complications. Created in BioRender. Avner, S. (2025) https://BioRender.com/2k45h24.

This regulation, known as glucose homoeostasis, is regulated primarily by hormones insulin and glucagon. Insulin promotes glucose uptake into muscles and adipose tissue, thereby reduce blood glucose levels after meals and glucagon acts on the liver, stimulating conversion of the liver’s glycogen stores to glucose, raising blood glucose levels for example in a fasting state or between meals.^5^ These processes are illustrated in Figure 1.

Glycaemic variability (GV) refers to blood glucose oscillations throughout the day. This can be postprandial hyperglycaemic episodes (also referred to as (“glucose spikes”), or hypoglycaemic episodes. GV is a physiological result of both carbohydrate intake and the normal circadian rhythm of hormones. However, when these fluctuations become excessive in amplitude or frequency, GV can become pathological. It is suggested that increased GV contributes to oxidative stress, endothelial dysfunction and inflammation – mechanisms associated with development of cardiovascular disease and type 2 diabetes.^7^

Oxidative stress is a cellular response arising from an imbalance between reactive oxygen species (ROS) and antioxidant defences, whereby excess ROS and/or insufficient antioxidant activity leads to oxidative damage.^8,9^ Although ROS at low to intermediate levels play important physiological roles such as cell signalling and immune defence,^10^ excessive accumulation can be harmful.^8^ ROS are generated by normal cellular metabolic processes as well as in response to hyperglycaemia and inflammation.^11,12^

Antioxidants are chemical substances that inhibit or slow oxidation of other molecules, thereby reducing oxidative stress.^12^ When antioxidant defences are overwhelmed or underactive, excessive ROS can induce DNA damage and activate proinflammatory pathways. They also modify proteins and lipids leading to the formation of advanced glycation end-products (AGEs) which in turn amplify inflammatory signalling, perpetuating further oxidative damage and inflammation.^9^

These processes are implicated in the pathogenesis of several chronic diseases such as cancer, cardiovascular disease and autoimmune conditions.^10^ Common biomarkers used to assess oxidative stress and ROS-induced damage include 8-hydroxy-2′-deoxyguanosine, Nitrotyrosine,^9^ and γ-H2AX.^13^

One key cardiovascular consequence of oxidative stress is atherosclerosis. Atherosclerosis is the formation of plaques primarily in the arterial vasculature due to endothelial injury and accumulation of lipids. ROS and AGEs damage the vascular endothelium, promote LDL oxidation and trigger inflammatory signalling, thereby contributing to the atherosclerotic process. Over time, this leads to narrowing of blood vessels, increasing the risk for strokes and myocardial infarction.^14^

Several physiological factors can exacerbate these pathways. Chronic stress causes activation of the HPA axis and increased cortisol production which induces muscle atrophy (sarcopenia) and infiltration of fat into muscle (myosteatosis), resulting in mitochondrial dysfunction and low grade inflammation.^15^ Sarcopenia is associated with an increase in AGEs which contribute to metabolic dysregulation including impaired blood glucose regulation.^16^

Similarly, a higher body mass index (BMI) is associated with chronic oxidative stress through increased levels of free fatty acids, adipose tissue inflammation and reduced antioxidant defences.^17^ Obesity is considered a state of low grade inflammation in which enlarged adipocytes secrete pro-inflammatory cytokines such as TNF-α further amplifying insulin resistance and oxidative stress.^18^

There is increasing interest, particularly from industry, around looking at glycaemic variability and glucose spikes in people without diabetes.^19,20^ CGM has become a popular topic and trend for people without diabetes,^21^ with some sources describing a “widespread obsession”^22^ with glucose levels, and “hacks”^22^ such as drinking vinegar before meals, or eating fats alongside sugar to reduce glucose spikes,^22^ being shared online. It is unclear whether CGM is beneficial for long-term health outcomes in people without diabetes. One study on CGM profiles in people without diabetes found the median time spent within the determined normal range for blood glucose of 3.9 to 7.8 was 96%.^23^ This helps to understand that despite inevitable post-prandial glucose spikes, people studied were still within the determined healthy range for glucose levels, therefore – this poses the question of do glucose spikes affect overall health in people without diabetes?

One factor that contributes to differences in glucose metabolism is the microbiome. The microbiome is made up of a variety of microorganisms including bacteria, archaea, fungi, protozoa and viruses. This community of microorganisms is essential for the normal host physiology such as metabolism, energy homoeostasis, immune function and gut epithelial health. Several factors can influence the microbiome and its normal function including diet, disease states and medical treatments.^24^

The microbiome has been shown to have a role in regulating blood glucose levels in response to food.^25^ This is because the bacteria within the microbiome produce short-chain fatty acids from dietary fibre, which in turn regulate insulin secretion, Glucagon-like Peptide hormone release, inflammation and energy metabolism.^26^

When the normal microbiome population is disrupted, this is called dysbiosis.^27^ Changes in the composition of the microbiome such as reduced abundance of butyrate-producing bacteria are associated with impaired glucose tolerance, even before diabetes is diagnosed.^28^ This highlights the importance of considering the microbiome when evaluating glucose spikes and their potential effects.

Given the impact of glucose spikes on healthy people without diabetes is a recently emerging idea, there is only a small evidence base. It is important to understand if CGM can benefit healthy individuals, or if it could inadvertently do more harm such as creating an obsessive culture around food and encouraging disordered eating behaviours. Therefore the current standpoint in the medical literature will be evaluated, and compared with the information shared with the general public through grey literature such as blogs, magazines and newspapers.

A systematic review and meta-analysis published in April 2024 by Hjort et al looked at the relationship between glycaemic variability in “individuals without diabetes and associations with cardiometabolic risk factors.”^29^ The differences in the formal and grey literature are not considered, therefore this project builds on this foundation piece to summarise what is currently known in this field, and to understand whether grey literature available to the general public aligns with medical research.

Key terms used throughout this article are defined in Table 1 (Table 1).

**Table 1.** Glossary for Key Terms.

Scientific term	Definition	Relevance to glycaemic control
Advanced glycation end-products (AGEs)	Harmful compounds formed when glucose reacts with proteins, lipids or nucleic acids	Accumulate with repeated glucose spikes, driving oxidative damage and risk of chronic diseases
Endothelial dysfunction	Impaired function of the endothelium (inner lining of blood vessels)	A consequence of oxidative stress and AGEs, an early step in atherosclerosis and cardiovascular disease
Glucose homoeostasis	The process of maintaining glucose levels within a narrow range – controlled by an interplay of hormones and metabolic processes	Disruption of the ability to maintain blood glucose within a narrow range can lead to hypo or hyperglycaemia, potentially predisposing to diabetes mellitus.
Glucose intolerance	Metabolic conditions where glucose homoeostasis is impaired, leading to higher glucose levels and predisposing to type 2 diabetes	Indicates impaired glucose regulation and can be a precursor to diabetes.
Glycaemic variability	Variation in glucose levels throughout the day	Linked to oxidative stress, inflammation and potential long-term complications.
Glycation	A chemical reaction where glucose binds to proteins, lipids or nucleic acids to form advanced glycation end-products	Glycation reactions increase during glucose spikes, AGEs contribute to oxidative stress, inflammation and vascular damage.
Insulin deficiency	Insufficient production of insulin	Lack of insulin impairs glucose uptake and promotes hyperglycaemia.
Insulin resistance	Insufficient response of cells to insulin	Reduces the body’s ability to respond to insulin, impairing glucose uptake by cells which leads to elevated postprandial and fasting glucose.
Oxidative stress	A state in which production of reactive oxygen species (ROS) exceeds the capacity of the body’s antioxidant defences leading to cellular and molecular damage.	Glucose spikes increase ROS, contributing to inflammation, endothelial dysfunction and insulin resistance.
Post-prandial glucose	Glucose levels 1-2 hours after a meal	Spikes in postprandial glucose reflect glycaemic variability.
Reactive oxygen species (ROS)	Highly reactive molecules derived from oxygen that can damage DNA, proteins and lipids	Overproduction from glucose spikes causes cellular damage, leading to inflammation and metabolic dysfunction.

Definitions reflect established physiological knowledge. Citations supporting related mechanisms are included in the main text.

## Aims and Objectives

This scoping review aims to compare the formal published literature with the grey literature with regards to glucose spikes and glycaemic variability in people without diabetes.

Objectives:

1. To review and compare the existing formal published literature and grey literature in order to understand what is known in the field of glycaemic variability for people without diabetes
2. Determine gaps in the knowledge
3. Summarise the data gathered and make recommendations on future research and potential for incorporation into clinical practice

## Methods

### Study Design

A scoping methodology was chosen to map current evidence in the formal literature and compare it with the grey literature. This approach enables detailed exploration, identification of knowledge gaps and recommendations for future research and practice.^30^ This review is guided by Arksey and O’Malley’s framework^30^ and advancements by Levac et al^31^ for scoping reviews. The PRISMA-ScR checklist^32^ ensured methodological consistency. The total duration of the study was 8 weeks.

The project was not registered with PROSPERO,^33^ as it does not accept scoping reviews. Ethical approval was not required.

### Search Strategy

For the formal literature, Medline and Embase (Ovid) were searched to cover medical, biomedical and pharmacological studies. Scoping searches were performed to optimise the search. Finalised on 13/10/2024, the search used the terms: glucose spike*; glucose oscillation*; glucose peak*; glycaemic variability; oscillating glucose AND non-diabet*; healthy people; people without diabetes OR endothelial cells; human endothelial cells, to maximise results both in humans or studies on human cells due to the limited number of studies on humans.

**Figure 2.**
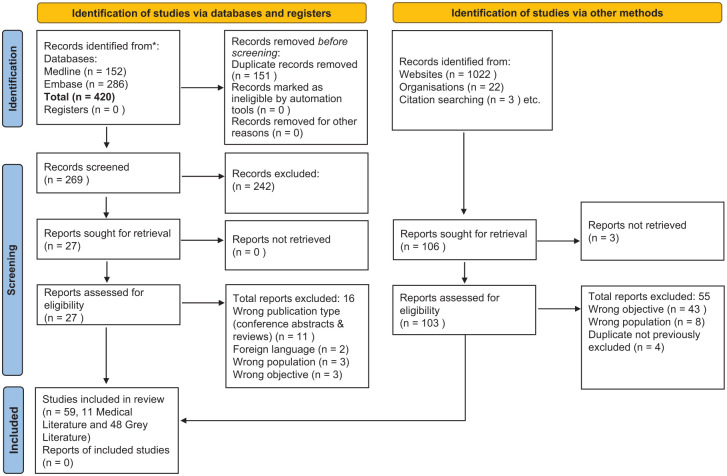
PRISMA flow diagram.

ProQuest database was searched for the grey literature. It is recognised as a key source of grey literature^34^ and was chosen due to its extensive coverage. The search, finalised on 13/10/2024, used key terms *(glucose spike OR glucose peak)* AND *(healthy people OR non-diabetic OR people without diabetes)* and filtered to sources such as magazines, newspapers, blogs and websites (Figure 2).

The search strategies are detailed in Appendices 1 and 2.

### Study Eligibility Criteria

Population: people without diabetes and human endothelial cells

Concept: the effect of glucose spikes on health (health described as any acute or long-term effects which stray from normal physiology and absence of disease).

Context: global studies and grey literature.

Inclusion and Exclusion Criteria can be found in Tables 2 and 3 below.

**Table 2.** Medical Literature Inclusion and Exclusion Criteria.

Inclusion criteria	Exclusion criteria
Available in English on Medline or Embase	Social media
Primary studies including random controlled trials, cohort studies, case control studies, cross sectional studies, case reports	Not available in English
Studies reporting the effect of glycaemic variability/glucose spikes on health for people without diabetes	Systematic Reviews and Meta-analyses
Studies on Humans	Studies focusing on diabetes exclusively
	Animal studies

**Table 3.** Grey Literature Inclusion and Exclusion Criteria.

Inclusion criteria	Exclusion criteria
Available in English	Social media
Source type: magazine, newspaper, blog, website	Conference Abstracts, Working Papers, Dissertations and Theses, any other grey literature that is not the source types specified in inclusion criteria
Reporting the effect of glucose spikes/glycaemic variability on health for people without diabetes	Not available in English
Report effect on humans	Sources focusing on diabetes exclusively
	Animal studies

Conference abstracts and other grey literature types were excluded as we wanted to compare sources readily accessible by the public with medical research findings.

### Screening and Data Management

Search results were imported into EndNote 20^35^ for de-duplication using a University of Warwick tool (Appendix 3). Data was then transferred to Rayyan^36^ for screening. Titles and abstracts were reviewed based on the eligibility criteria, followed by full-text reviews of remaining studies.

In the grey literature, several organisations who are key players in the CGM industry were mentioned. The websites of these organisations were checked for an in-house blog, and any relevant blog posts were included.

### Data Charting

Two Excel data extraction sheets were developed. They were piloted with 2 formal and 2 grey literature sources. The 2 data extraction sheets are slightly different from one another to account for the different nature of the sources – the grey literature lacks scientific headings such as outcomes and objectives, therefore required adaptation.

Studies were extracted with their characteristics such as location, aims and objectives, study design and methods. Outcome measures were extracted as; primary outcomes, secondary outcomes, impact of glucose spikes and overall themes and conclusions.

## Results

### Study Demographics

45.5% of studies were from Italy, 27.3% from the UK and the remainder from other countries including the USA, Turkey and Romania. 63.6% of studies were of controlled experimental design on human endothelial cells. The remaining 36.4% was split into controlled experimental design on humans and cohort studies (Table 4).

**Table 4.** Study Locations and Study Design for Formal Literature.

Author	Year	Study location	Study design
Ceriello et al^37^	2008	UK	Controlled Experimental (Humans)
La Sala et al^46^	2016	Italy	Controlled Experimental (Human Endothelial Cells)
La Sala et al^45^	2018	Italy	Controlled Experimental (Human Endothelial Cells)
La Sala et al^38^	2015	Italy	Controlled Experimental (Human Endothelial Cells)
Liang et al^49^	2016	USA	Observational Cohort
Piconi et al^43^	2004	Italy	Controlled Experimental (Human Endothelial Cells)
Quagliaro et al^44^	2005	Italy	Controlled Experimental (Human Endothelial Cells)
Schisano et al^47^	2011	UK	Controlled Experimental (Human Endothelial Cells)
Sezer et al^51^	2021	Turkey	Cohort
Toma et al^48^	2023	Romania	Controlled Experimental (Human Endothelial Cells)
Wakil et al^50^	2012	UK	Controlled Experimental (Humans)

The formal literature consisted of 5 Italian studies, 3 UK studies, 1 Turkish study, 1 Romanian study and 1 study from the USA. The grey literature consisted of 25 sources from the USA, 18 from the UK, 2 from India, 1 from Finland, 1 from Israel and 1 from Canada (Table 5).

**Table 5.** Comparing Country of Origin.

Country of origin	Formal literature	Grey literature
USA	1	25
UK	3	17
Italy	5	0
Turkey	1	0
Romania	1	0
India	0	2
Israel	0	1
Canada	0	1
Finland	0	2

The grey literature was comprised of 16 magazine articles, 12 newspaper articles, 20 blogs and 1 website (Table 14 in Appendix 4).

## Medical Literature

### Effect on the Endothelium

The literature found that glucose spikes induce endothelial dysfunction, measured by flow-mediated dilation of the brachial artery.^37^ Additionally, OG increased endoglin expression,^38^ a glycoprotein involved in angiogenesis and vascular remodelling.^39^ Angiogenesis refers to the formation of new blood vessels from pre-existing vasculature, and is involved in tissue growth and repair.^40^ However, uncontrolled angiogenesis underlies many pathological conditions such as cancer and autoimmune conditions.^41^ Vascular remodelling refers to the molecular and structural alterations in the vasculature that result from endothelial cell injury, inflammation and cardiovascular disease.^42^

OG also increased expression of adhesion molecules ICAM-1, VCAM-1 and E-selectin, which promote atherosclerosis^43,44^ (Table 6).

**Table 6.** Effects of Oscillating Glucose on the Endothelium.

Author	Year	Effect
Ceriello et al^37^	2008	Endothelial dysfunction
La Sala et al^38^	2015	Expression of endoglin
Piconi et al^43^	2004	Expression of ICAM-1, VCAM-1, E-selectin
Quagliaro et al^44^	2005	Expression of ICAM-1, VCAM-1, E-selectin

Abbreviations: ↑: increased; ICAM-1: intercellular adhesion molecule 1; VCAM-1: vascular cell adhesion molecule 1.

### Effect on Oxidative Stress

#### Upregulation of Oxidative Stress Factors

The literature indicates that both OG and HG levels upregulated miR-21 and oxidative stress markers – superoxide anion, 8-OHdG, and γ-H2AX – with no difference between OG and HG conditions.^45^ La Sala et al and Schisano et al both found that OG did cause a greater increase in γ-H2AX than constant HG, and levels remained elevated after 1 week of glucose normalization^46,47^

Proteins associated with oxidative stress regulation, including PKC delta, ROS, p53, PTEN, p21, PUMA and TIGAR, were significantly upregulated in OG compared to HG and remained elevated after normalisation.^47^

Additionally, ROS, VPO-1, and p22phox were higher under OG than normal or HG conditions.^48^ OG conditions also more than doubled 8-OHdG levels and significantly increased PKC isoform activity (b1, b2, delta) after 14 days.^44^ Markers of oxidative stress, Nitrotyrosine and 8-iso PGF2a, were also elevated under OG conditions compared to others^37,43^ (Table 7).

**Table 7.** Effects of Oscillating Glucose on Oxidative Stress: Upregulated Factors.

Author	Year	(↑) (↓)	Effect
La Sala et al^45^	2018	(↑) (↑) (↑) (↑)	miR-21 superoxide anion, 8-OHdG γ -H2AX
La Sala et al^46^	2016	(↑)	γ -H2AX
Schisano et al^47^	2011	(↑) (↑) (↑) (↑) (↑) (↑) (↑) (↑)	γ -H2AX PKC δ ROS p53 PTEN p21 PUMA TIGAR
Toma et al^48^	2023	(↑) (↑) (↑)	ROS VPO-1 p22phox
Quagliaro et al^44^	2005	(↑) (↑)	8-OHdG PKC (β1, β2, δ)
Ceriello et al^37^	2008	(↑) (↑)	Nitrotyrosine 8-iso PGF 2α
Piconi et al^43^	2004	(↑)	Nitrotyrosine

Abbreviations: ↑: increased; ↓: decreased; miR-21: microRNA-21; 8-OHdG: 8-hydroxyguanosine; γ -H2AX: gamma H2A histone family member X; PKC δ: protein kinase C delta; p53: tumour protein 53; PTEN: phosphatase and tensin homologue; p21: cyclin-dependent kinase inhibitor 1; PUMA: p53 upregulated modulator of apoptosis; TIGAR: TP53-induced glycolysis and apoptosis regulator; ROS: reactive oxygen species; VPO-1: vascular peroxidase 1; 8-iso-PGF 2a: 8-iso-prostaglandin F2α.

#### Downregulation of Anti-Oxidants

The literature also found that both OG and HG upregulated miR-21, which coincided with downregulation of anti-oxidant genes SOD-2 and KRIT-1.^45^ OG also caused more significant downregulation of miR-185 than constant HG, which led to reduced levels of anti-oxidant GPx-1^46^ (Table 8).

**Table 8.** Effects of Oscillating Glucose on Oxidative Stress: Downregulated Factors.

Author	Year	(↑) (↓)	Effect
La Sala et al^45^	2018	(↓) (↓)	SOD-2 KRIT-1
La Sala et al^46^	2016	(↓) (↓)	GPx-1 miR-185

Abbreviations: ↑: increased; ↓: decreased; SOD-2: superoxide dismutase 2; KRIT-1: Krev interaction trapped protein 1; GPx-1: glutathione peroxidase 1.

### No Significant Association

Liang et al looked at the associations between urinary 1-5 anhydroglucitol (low levels indicate hyperglycaemia) levels and high-sensitivity cardiac troponin T (Hs-Ctnt), a marker of subclinical myocardial damage. They found no association between low levels of urinary 1-5 anhydroglucitol, and Hs-Ctnt in people without diabetes, providing limited evidence of any association between glucose spikes and adverse cardiovascular outcomes in this population.^49^

Wakil et al investigated the effect of normal glucose, HG and OG on oxidative stress marker urinary 8-iso-PGF2a and found no significant difference in urinary 8-iso-PGF2a in healthy subjects following the 3 glycaemic states.^50^

### Inflammation

Toma et al found that OG caused statistically significant upregulation of the NINJ-1 gene, and silencing NINJ-1 reduced the effects of OG on endothelial function. Additionally, expression of inflammatory markers MCP-1, TNFR1, RAGE, VAMP-3, NF-kB and p38 MAPK were also significantly upregulated by OG. OG also stimulated adhesion of monocytes to endothelial cells to a greater extent than HG^48^ (Table 9).

**Table 9.** Effects of Oscillating Glucose on Inflammation.

Author	Year	(↑) (↓)	Effect
Toma et al^48^	2023	(↑) (↑) (↑) (↑) (↑) (↑) (↑) (↑)	NINJ-1 MCP-1 TNFR1 RAGE VAMP3 NF-kB p38 MAPK Monocyte adhesion

Abbreviations: ↑: increased; ↓: decreased; NINJ-1: Ninjurin-1; MCP-1: Monocyte Chemoattractant Protein-1; TNFR1: tumour necrosis factor receptor 1; RAGE: Receptor for Advanced Glycation End-products; VAMP3: Vesicle-associated Membrane Protein 3; NF-kB: Nuclear Factor kappa-light-chain-enhancer of activated B cells; p38 MAPK: p38 Mitogen-Activated Protein Kinase.

### Blood Pressure

Sezer et al investigated the association between glycaemic variability and blood pressure variability. A statistically significant correlation was found between daytime BPV parameters, and all markers of blood glucose.^51^ See Table 10 for description of this study.

**Table 10.** Summary of Sezer et al Study.

Study ID	Population	No. participants	Age distribution	Gender distribution F/M	Parameters measured	Statistical methods
Sezer et al 2021^51^	Healthy normotensive, normo-glycaemic individuals	27	23.8 ± 2.7 y	18/9	BP: measured every 15 min during day and 30 min at night. BP Parameters: 24h, daytime, nighttime SBP/DBP (mean, SD) Blood glucose measured every 15 min. GV parameters: MBG, SD, MAGE, MODD, CV, day/night average glucose	Descriptive statistics (mean ± SD), normality testing, correlation analysis using Pearson/Spearman tests

Abbreviations: BP: blood pressure; SBP: systolic blood pressure; DBP: diastolic blood pressure; GV: glycaemic variability; MBG: mean blood glucose; MAGE: mean amplitude of glycaemic excursions; MODD: mean of daily differences; CV: coefficient of variability of blood glucose.

## Grey Literature

### Hunger

Grey literature sources commonly associate glucose spikes with increased hunger and cravings, often attributing this to rapid changes in insulin and blood glucose levels.

The grey literature described that glucose spikes cause hunger,^52-57^ causing people to eat more, triggering an “unhealthy cycle.”^58^ It was found that that glucose spikes cause cravings,^54-56,59-61^ with one source stating if you start the day with sweet foods, “you’ll have cravings every two hours until you go to bed”.^62^

Several sources commented that high insulin levels in response to glucose spikes cause a rapid decline in blood glucose levels, inducing a state of hunger.^63-67^ One source describes the insulin fluctuation as having a “drug-like effect,”^68^ causing one to eat more to “get high again.”^68^

Some sources reported glucose spikes induce thirst.^52,55,69^

### Fat Storage

Fat storage is frequently linked to glucose spikes in grey literature sources, with several citing that elevated insulin levels or reduced fat burning as contributing factors.

The grey literature suggests high insulin levels from glucose spikes promote fat storage^53,63,64^ One source links glucose fluctuations to weight gain via appetite-regulating hormones,^59^ while another attributes it to “carb snacking”^57^ and reduced fat burning, causing weight gain.^57^ Other sources note glucose spikes leading to fat storage without specifying a cause.^65,66,70,71^

### Skin Health

Some sources propose that glucose fluctuations may accelerate ageing or cause acne, referencing glycation and changes in collagen structure.

Grey literature sources discussed that fluctuations in glucose have an adverse effect on skin,^54,60,72^ speed up skin ageing,^73^ and can cause acne.^59^ Glucose spikes are linked to glycation, which weakens collagen, making it less elastic and leading to “fine lines, wrinkles and sagging.”^74^ Another source discussed that being exposed to high blood sugar frequently can cause collagen molecules to cross-link, making skin stiffen and look “leathery.”^73^

### Mental Health

The grey literature links glucose spikes to mood swings, anxiety and depressive symptoms, occasionally suggesting these mechanisms may be via blood sugar crashes or hormonal stress responses such as the release of adrenaline and noradrenaline.

Glucose spikes have been associated with increased risk of depression^54,71,75^ and anxiety,^56,59,71,75^ “feelings of sadness,”^56^ mood swings and irritability.^54^ One source explained that the increase in insulin following a glucose spike can cause blood sugar levels to drop below where they started, causing release of adrenaline and noradrenaline to help raise blood sugar again, but potentially causing anxiety, fear and aggression as a byproduct.^75^ Another source described that the brain is sensitive to glucose spikes, and this has been linked to anxiety and depression in people with a medical history of them.^62^

### Energy and Mood

Glucose spikes are described as affecting energy levels and concentration, often in the form of rapid energy highs followed by fatigue or “crashes.”

Several sources noted that glucose spikes lead to difficulty concentrating^52,54,60,71,76-78^ and less energy.^52,54,56,60,76,79,80^ One source described sugary foods as “bad mood foods” due to the rush of energy they give which culminates in an energy slump.^81^ Additionally, with respect to nutrition, a high glycaemic index (GI) diet – a diet that causes more glucose spikes^82^ – is associated with lower mental alertness than a low GI diet.^65^ Fatigue was also highlighted as a key effect of glucose spikes,^52,53,60,61,67,69,71,73,78,83^ as well as brain fog.^53,55,60-62,69,73,77^

Two sources described authors’ personal experiences in reducing glucose spikes and removing sugar from their diet. They describe that they have more energy, better sleep, less mood swings, less tiredness^84,85^ and that they “feel lighter, free of rampaging hunger”^84^ and that “running in the mornings has become a delight.”^84^

Mood swings and a negative impact on mood was reported as an effect of glucose spikes,^52,54,59,76,85,86^ and one source described that glucose spikes and crashes can feel like an “emotional rollercoaster.”^87^

Irritability^55,56,62^ and feeling lightheaded, sweating or shaking^61,67^ were reported as other effects of glucose spikes. One article described it as a “crashing feeling,”^83^ leading to shaking, irritability, palpitations, and fatigue.”^83^ Another article described foods causing glucose spikes as putting blood sugar on a “rollercoaster ride,”^67^ leading to the sensation of being on a rollercoaster, causing one to feel “exhausted, dizzy and sick.”^67^

### Sleep

Several sources suggest that glucose spikes impair sleep, some of which reference cortisol release or disruptions in glucose regulation overnight.

The grey literature explains that glucose spikes are linked to lower quality of sleep.^52,54,59,76,86^ In response to glucose spikes, the body may produce higher levels of cortisol and adrenaline which can hinder sleep at night. This is reported as a vicious cycle, as lack of sleep can boost blood sugar, increase appetite and lead to further increase in cortisol and adrenaline.^73^

### Memory

Two sources report glucose spikes affecting memory.^67,88^ One source reports that a 2008 study found glucose spikes lead to reduced blood flow in part of the hippocampus involved in memory formation.^65^

### Myopia

Myopia refers to a refractive error of the eye, where light entering the eye is focused in front of the retina rather than directly on it – causing difficulty seeing distant objects clearly (short-sightedness).^89^ One article reports researchers found insulin spikes caused by glucose spikes in childhood may disrupt normal eye development and contribute to myopia.^84^

### Mortality Risk

Mortality risk refers to the likelihood of death within a defined time period. One source claimed researchers have linked glucose spikes to increased mortality risk in people without diabetes,^90^ though mechanisms are not discussed in detail.

### Glycaemic Variability

Several sources acknowledge that a degree of glycaemic variability is normal,^52,91-94^ reflecting natural responses to food intake and hormonal rhythms, and that many healthy people without diabetes will not feel acute effects of glucose spikes.^80^

### Inflammation and Glycation

Grey literature frequently links glucose spikes to inflammation and glycation, describing effects such as cellular damage, endothelial dysfunction and increased risk of disease.

Glucose spikes can trigger glycation^74,95^ through chronic inflammation,^95^ producing harmful molecules called advanced glycation end products.^74,94^ These damage cells, stiffen blood vessels via assisting plaque formation^94,95^ and can contribute to diabetes, heart disease,^74,95^ and stroke.^95^

Persistent high blood sugar can promote inflammation^53,74,96^ via a surge in free radicals,^73^ which can damage the lining of blood vessels^53,73^ cause blood clots,^73^ and can exacerbate skin conditions such as eczema and psoriasis.^74^

One source suggested glucose spikes cause inflammation and trigger mitochondrial stress, leading to exhaustion, poor sleep and sugar cravings.^62^

One source describes that glucose spikes indirectly cause inflammation, and that this can increase the risk of type 2 diabetes, heart disease and some cancers, as well as causing increased fat storage, which leads to further inflammation.^97^

High blood glucose causes oxidative stress, producing excess free radicals, leading to inflammation and damage to cells.^98^ One source stated that an uncited study on healthy participants found raised inflammatory markers when researchers artificially increased their blood glucose levels.^94^

### Other Effects

Various other effects of glucose spikes were mentioned in the grey literature (Table 11).

**Table 11.** Other Effects of Glucose Spikes – Grey Literature.

Author	(↑) (↓)	Effect of glucose spikes
Dunn, 2007^85^	(↓) (↑) (↑)	Immune system Risk of cardiovascular disease Risk of osteoporosis
Wilson, 2024^86^	(↓)	Weight loss
Jibrin, 2001^65^	(↑) (↑) (↑) (↑) (↓)	Risk of cardiovascular disease Colon cancer Blood pressure Triglycerides High Density Lipoprotein
Anonymous, 2013^66^	(↓)	B vitamin absorption
Gower, 2000^99^	(↓)	High Density Lipoprotein
Davis, 2024^92^	(↑) (↑) (↑) (↑)	Risk of cardiovascular disease Risk of Alzheimer’s Disease Risk of certain cancers Priming antioxidant defence mechanisms to offset damage of glucose spikes
Eckelkamp, 2024^71^	(↓)	“Workout performance”
Migala, 2023^69^	(↑)	Headaches
Larsen, 2022^100^	(↑)	Risk of cardiovascular disease

### Insulin Resistance and Diabetes

The grey literature found that glucose spikes lead to insulin resistance^96,99^ and diabetes^96,99,100^ with one source saying that large spikes can predispose to pre-diabetes^79^ and another describing that glucose spikes increase the risk of “glucose intolerance”,^85^ a precursor for type 2 diabetes.^85^

### Effects of Repeated Glucose Spikes

Many sources in the grey literature highlighted that the effects of glucose spikes are cumulative, and that frequent glucose spikes over a long period of time is more harmful than isolated events (Table 12).

**Table 12.** Effects of Repeated Glucose Spikes – Grey Literature.

Author	Effect of glucose spikes
Matei, 2024^78^	Insulin Resistance leading to pre-diabetes and type 2 diabetes
Bede, 2023^54^	Insulin Resistance leading to pre-diabetes and type 2 diabetes Increased risk of cardiovascular disease
Givens, 2023^55^	Insulin Resistance leading to pre-diabetes and type 2 diabetes Increased risk of cardiovascular disease
Zakrzewski, 2022^91^	Insulin Resistance leading to pre-diabetes and type 2 diabetes Increased risk of cardiovascular disease Weight gain
ZOE, 2020^61^	Insulin Resistance leading to pre-diabetes and type 2 diabetes Increased risk of cardiovascular disease Increased testosterone levels leading to Polycystic Ovarian Syndrome
Eckelkamp, 2024^71^	Insulin Resistance leading to pre-diabetes and type 2 diabetes Increased risk of cardiovascular disease Increased risk of cancer Increased risk of kidney disease Increased oxidative stress
Newman, 2024^94^	Insulin Resistance leading to pre-diabetes and type 2 diabetes Increased risk of cardiovascular disease Increased reactive oxygen species & oxidative stress
Levels, 2023^56^	Insulin Resistance leading to pre-diabetes and type 2 diabetes Increased risk of cardiovascular disease Increased risk of dementia Poorer memory in young adults Infertility Increased risk of cancer Increased mortality risk Damage to blood vessels → problems with blood flow/pressure → stroke Increased oxidative stress
Montague, 2003^101^	Insulin Resistance leading to obesity, high blood pressure, raised cholesterol
Markman, 2024^57^	Insulin Resistance
Piore, 2023^95^	Increased risk of diabetes
Jibrin, 2001^65^	Increased risk of diabetes Increased risk of cardiovascular disease Increased risk of cancer
January AI, 2021^80^	Increased risk of diabetes Increased risk of cardiovascular disease
Levels, 2023^60^	Weight gain Hormonal dysfunction Increased risk of dementia Increased risk of cardiovascular disease
De Lange, 2022^79^	Increased risk of dementia Lethargy Ageing of skin Damage to blood vessels → Increased risk of cardiovascular disease Increased risk of cancer
Koenck, 2023^76^	Damage to blood vessels leading to: Increased risk of cardiovascular disease Increased inflammation Risk of kidney disease Poor wound healing
Davis, 2024^92^	Higher risk of disease generally
Corindia, 2024^93^	Higher risk of disease generally

Below is a comparison table of the results from the medical and grey literature (Table 13).

**Table 13.** Comparison of Medical and Grey Literature Findings.

Topic	Medical literature	Grey literature
Hunger and thirst	Not reported in medical literature findings.	Glucose spikes cause hunger and cravings and induce thirst.
Fat storage	Not explicitly addressed in medical literature findings	Glucose spikes promote fat storage and weight gain.
Skin health	Not reported in medical literature findings.	Glucose spikes speed up ageing, cause acne, can cause fine lines, wrinkles and sagging due to weakening of collagen.
Mental health	Not reported in medical literature findings.	Glucose spikes associated with increased risk of depression and anxiety, mood swings and irritability.
Energy/mood	Not reported in medical literature findings.	Glucose spikes lead to difficulty concentrating, lower energy, fatigue and brain fog.
Sleep	Not reported in medical literature findings.	Glucose spikes are linked with lower quality of sleep.
Memory	Not reported in medical literature findings.	Glucose spikes reduce hippocampal blood flow.
Myopia	Not reported in medical literature findings.	Glucose spikes can disrupt normal development of eyes leading to myopia.
Mortality risk	Not reported in medical literature findings.	Glucose spikes increase general mortality risk.
Inflammation/Glycation	Markers of oxidative stress were upregulated, and anti-oxidants were downregulated in oscillating glucose conditions	Glucose spikes increase oxidative stress and AGEs, leading to inflammation and endothelial dysfunction
Insulin resistance/diabetes	Repeated glucose spikes may contribute to insulin resistance and progress to type 2 diabetes	Chronic glucose spikes promote insulin resistance which can progress to type 2 diabetes

## Discussion

It is noticeable that there are substantial differences between country of origin of the formal and the grey literature. The majority of grey literature sources originated from the USA, compared with only one paper from the medical literature. This may be due to the more commercialised health sector in the USA.^102^

63.6% of the medical literature (6 out of 11 papers) use endothelial cells, making it clear that there is little research on the effect of glucose spikes in human subjects specifically.

It is known that repeated glucose spikes that is, a prolonged state of hyperglycaemia can cause insulin resistance,^103^ which contributes to the development of diabetes,^104^ as the grey literature also describes. However, the primary aim of this article is to determine whether glucose spikes are detrimental to the health of people without diabetes.

Both the medical and grey literature agree that glucose spikes can cause endothelial dysfunction, oxidative stress and inflammation. The grey literature characterises this as damage to blood vessels, leading to cardiovascular disease, poor wound healing, damage to the kidneys and increased blood pressure.

However, the grey literature makes additional claims, not found in the search of the medical literature. These include effects on hunger, cravings, mental health, energy and mood, memory, hormonal dysfunction, weight gain and cancer risk.

The discrepancies between the medical and grey literature could be problematic. The grey literature may be sharing information that is not scientifically proven and published through traditional peer reviewed routes, and this could lead to confusion amongst readers, perhaps even leading to disordered eating behaviours.^22^

One source describes one of the key players in the CGM industry for healthy individuals has “reached almost cult status,”^105^ calling it “expensive. . .appealing and addictive.”^105^ This highlights it may have become trendy to trial these products, but if glucose levels are within normal range, do these spikes matter? Another source states there aren’t many studies relevant specifically to people without diabetes, and information not backed by scientific data is shared, causing distress to people – which may be worse for their health than the spikes themselves.^86^ This aligns with our results, where only 11 medical studies were found, compared to many more grey literature sources.

Some sources argue CGM is unnecessary for people without diabetes who are within normal glucose ranges, and that knowing our glucose levels at all times could “amount to splitting hairs,”^78^ and that “unless you have diabetes, your body is well-equipped to handle this [glucose] through insulin production.”^22^ Another source explaining that glucose levels in people without diabetes is a small part of one’s overall health.^106^

CGM has the clear benefit of allowing one to monitor their blood glucose, however, it may encourage “glucose-centricity”^106^ – focusing on one parameter just because we can measure it – whereas there are many other factors that may affect our health that we aren’t currently able to measure.^106^

The microbiome varies from person to person,^107^ and has a significant impact on how the body responds to different foods.^108^ For example, some people may have a large glucose spike in response to ice cream, and others won’t. Therefore, generalised advice such as eating low GI may not be appropriate for everyone. There is therefore potential for personalised nutrition based on the microbiome in reducing glucose spikes, in turn reducing incidence of disease that may be caused by glucose spikes.

However, many studies within the field of personalised nutrition and CGM are cross-sectional, and find associations rather than causation, which can lead to generation of false positives,^106^ complicating the interpretation of results.

In the author’s opinion, further work is needed on leading CGM providers. Firstly, suggesting there are not yet enough peer-reviewed studies to prove the efficacy of using CGM in people without diabetes to improve their health. Secondly, one source claims that these organisations appeal to individuals who don’t require it – “health conscious and well-off”^105^ people who already look after their health and are already at lower risk of disease.^105^

One blog promoting CGM for people without diabetes advises that their message is not to avoid all carbohydrates and sugars – it is about choosing the right kinds for example, whole foods over processed foods.^109^ This is reassuring from a key player in the industry, as it appears as a more balanced viewpoint and is less likely to instil fear of certain foods onto people.

### Limitations

There are some limitations of the study to acknowledge. First, there was only one reviewer for screening and data extraction. Second, only one grey literature database was used. These are both due to time and resource constraints. Lastly, the data extraction tables differ slightly between the grey and formal literature, which was due to the different nature of the sources.

### Future Work

The grey literature suggests that some people experience changes in mood and energy levels – however these are subjective experiences, making it difficult to determine causation. Furthermore, most of the medical literature focuses on endothelial cells, therefore cannot investigate these subjective experiences.

In order to understand the potential health benefits of controlling glucose spikes in people without diabetes and for incorporation of this information into clinical practice, further research on human subjects is needed. Further research will also aid understanding of whether the grey literature has traditional equivalent evidence from peer-reviewed medical literature.

## Conclusion

Glucose spikes may impact the health of people without diabetes, but significant health outcomes are more likely a result of repeated long-term glucose spikes rather than isolated acute spikes. Discrepancies between the medical and grey literature highlight the potential for misinformation,^110^ although the author does not suggest any of the grey literature sources cited are misinforming the public. It is also important to note that the presence of information in the grey literature but not in the medical literature does not directly mean the grey literature content is misinforming. Further research would be valuable, as hypothetically, the impact of any misinformation on consumer wellbeing could be significant.

## Supplemental Material

sj-docx-1-end-10.1177_11795514251381409 – Supplemental material for A Scoping Review of Glucose Spikes in People Without Diabetes: Comparing Insights from Grey Literature and Medical Research Supplemental material, sj-docx-1-end-10.1177_11795514251381409 for A Scoping Review of Glucose Spikes in People Without Diabetes: Comparing Insights from Grey Literature and Medical Research by Shira Avner and Timothy Robbins in Clinical Medicine Insights: Endocrinology and Diabetes

## Appendix 1

Medline and Embase Search Strategy

## Appendix 2

Grey Literature Search Strategy

## Appendix 3

Warwick De-Duplication Tool

https://warwick.ac.uk/services/library/students/endnote/EndNote-de-duplication-table.pdf

We applied the following parameters: default, 1+, 8+ and 10+.

## Appendix 4

**Table 14.** Source Type for Grey Literature.

Author	Year	Source type
Webber^58^	2006	Magazine
Pearson^63^	2002	Magazine
Scourboutakos^75^	2024	Newspaper
Shallcross^64^	2005	Newspaper
Montague^101^	2003	Magazine
Anonymous^68^	2017	Newspaper
Evennett^87^	2001	Magazine
Gold^53^	2020	Magazine
Anonymous^81^	1999	Magazine
Anonymous^96^	2023	Newspaper
Piore^95^	2023	Magazine
Laliberte^73^	2016	Magazine
Ramsden^62^	2024	Newspaper
Geddes^97^	2022	Newspaper
De Lange^79^	2022	Magazine
Morris^90^	2021	Newspaper
Judson^84^	2019	Newspaper
Matei^78^	2024	Newspaper
Anonymous^74^	2014	Magazine
Dunn^85^	2007	Magazine
Wilson^86^	2024	Magazine
Anonymous^88^	2011	Newspaper
Jibrin^65^	2001	Magazine
Desanctis^83^	2011	Magazine
Anonymous^66^	2013	Newspaper
Gower^99^	2000	Magazine
VanTine^67^	2006	Magazine
Bede^52^	2023	Blog
Bede^54^	2023	Blog
Bede^72^	2023	Blog
Givens^55^	2023	Blog
Koenck^76^	2023	Blog
Tillman^70^	2022	Blog
Zakrzewski^91^	2022	Blog
Washuta^98^	2023	Blog
Davis^92^	2024	Blog
Larsen^100^	2022	Blog
Team TS^77^	2024	Blog
Jayawardene^59^	2024	Blog
Corindia^93^	2024	Website
Levels Team^56^	2023	Blog
Eckelkamp^71^	2024	Blog
Migala^69^	2023	Blog
Levels Team^60^	2023	Blog
Markman^57^	2024	Blog
January AI^80^	2021	Blog
Newman^94^	2024	Blog
ZOE^61^	2020	Blog
